# Predicting sleep apnea worsening and cardiometabolic risk without treatment

**DOI:** 10.1007/s11325-026-03617-y

**Published:** 2026-04-21

**Authors:** Peretz Lavie

**Affiliations:** Rappaport Faculty of Medicine, Technion – Israel Institute of Technology, Haifa, Israel

**Keywords:** Obstructive sleep apnea, Apnea progression, Longitudinal study, Cardiometabolic comorbidities, Sleep quality

## Abstract

**Purpose:**

The natural history of untreated obstructive sleep apnea (OSA) and its cardiometabolic consequences are not fully characterized. We aimed to identify predictors of apnea severity progression and incident cardiometabolic comorbidities in untreated patients, comparing continuous and categorical measures of apnea severity.

**Methods:**

We retrospectively studied 538 adults (419 men, 119 women) who underwent two attended overnight polysomnographic evaluations separated by 3.8 ± 2.7 years and declined OSA treatment. Changes in apnea severity were assessed using continuous change in apnea–hypopnea index (ΔAHI) and categorical transitions across standard severity thresholds. Multivariable linear regression was used to identify predictors of ΔAHI, and multivariable logistic regression was used to identify predictors of incident hypertension, diabetes, and cardiovascular disease.

**Results:**

Apnea severity and nocturnal hypoxemia worsened significantly over time. In linear regression, worsening ΔAHI was independently associated with higher baseline body mass index (BMI), greater weight gain, more severe baseline hypoxemia, and longer sleep latency, whereas higher baseline AHI was associated with smaller subsequent increases. In contrast, categorical progression to moderate or severe OSA was primarily predicted by baseline BMI. Incident hypertension and diabetes were independently associated with age, baseline BMI, and duration of follow-up, but not with baseline AHI or ΔAHI. Incident cardiovascular disease was independently associated with baseline apnea severity and lower sleep efficiency. Model discrimination ranged from acceptable to good (C-statistics 0.75–0.86).

**Conclusion:**

In untreated patients, OSA commonly worsens over time, with progression influenced by obesity, hypoxemia, and sleep quality. Continuous measures of apnea severity capture physiologic progression more sensitively than categorical classifications. Cardiometabolic consequences differ by outcome, with obesity predominating for hypertension and diabetes and apnea severity and sleep fragmentation more closely linked to cardiovascular disease.

## Introduction

 Obstructive sleep apnea (OSA) is a common chronic disorder characterized by recurrent upper airway collapse during sleep, leading to intermittent hypoxemia, sleep fragmentation, and significant neurocognitive and cardiovascular morbidity. Although treatments such as continuous positive airway pressure (CPAP) and upper airway surgery are well established, the natural history of untreated OSA remains insufficiently understood [1]. Understanding this trajectory is crucial, as untreated OSA contributes to substantial long-term health risks, including hypertension, arrhythmias, metabolic dysfunction, and mortality.

Early longitudinal studies have shown that OSA can worsen over time in untreated patients. Inoue et al. [2] demonstrated that over a five-year follow-up of Japanese patients, untreated individuals experienced significant deterioration in respiratory indices, including prolonged apneic episodes and increased nocturnal desaturation. These effects were most pronounced in middle-aged adults (40–60 years), independent of obesity, highlighting age as a key determinant of disease progression. In contrast, Fisher et al. [3] reported that patients evaluated 5 years apart did not show significant changes in apnea severity nor in body weight. There was, however, a significant correlation between the changes in body weight and the respiratory disturbance index.

Population-based studies, such as the Wisconsin Sleep Cohort, have confirmed that body weight plays a central role in modulating apnea severity. Peppard et al. [4] found that a 10% increase in body weight predicted a 32% rise in the apnea–hypopnea index (AHI), while a similar degree of weight loss led to a 26% reduction. Thus, obesity is a critical modifiable factor in the development and progression of OSA. Interestingly, even in patients with a stable body mass index (BMI), several studies have shown progressive worsening of AHI and nocturnal hypoxemia, suggesting that other factors—such as upper airway anatomy, neuromuscular tone, and age-related changes—also contribute to disease advancement [5].

Age-dependent trends have also been described. Bixler et al. [6] and Sforza et al. [7] reported that OSA tends to worsen during midlife, whereas older adults may experience stabilization or even improvement in disease severity. This divergence may reflect age-related changes in sleep architecture, ventilatory control, or airway collapsibility. Similarly, Lindberg et al. [8] found that middle-aged individuals with untreated OSA often show a progressive increase in apneic events and hypoxemia, while older cohorts may show attenuation of severity.

More recent studies have explored phenotype-specific factors. In the Sleep Heart Health Study, Aurora et al. [9] observed that individuals with rapid eye movement (REM)-predominant OSA often remained stable, whereas progression to non-REM OSA was more likely among women and those with higher BMI or advancing age. These findings suggest that sleep stage distribution and sex-specific factors influence disease progression and related cardiovascular risk.

Overall, the natural course of untreated OSA is heterogeneous and shaped by interacting variables such as age, baseline severity, obesity, and OSA phenotype. The evidence points to a general tendency toward progression in untreated middle-aged adults—often exacerbated by weight gain—contrasted with relative stability or improvement among older individuals. Understanding these processes is essential for identifying high-risk patients, optimizing monitoring strategies, and emphasizing early intervention.

Accordingly, this study aims to identify predictors of changes in sleep apnea severity and the development of comorbidities among untreated patients.

## Methods

### Study population

The Technion Sleep Disorders Center had 4 clinics in major cities in Israel. Between 2002 and 2024, 126,726 whole-night attended PSG recordings were performed, of which more than 71,000 were due to suspected OSA in adult patients. All clinics used the same equipment (Embla S4500) and scored PSG records with the help of the REMlogic software. Patients with a documented diagnosis of OSA were referred to treatment, mostly with CPAP. Between 2002 and 2024, 18,456 PSG-monitored CPAP titrations were performed. The present study included 538 patients (419 men and 119 women) referred because of suspected OSA, due to complaints of chronic fatigue, loud snoring, and witnessed apneas during sleep. These patients declined any treatment and because of persistent symptoms or incidental morbidities underwent a second attended PSG evaluation approximately 3.8 ± 2.7 years after the first. The two PSGs were conducted in the same way, and the same scoring criteria were applied and scored independently. None of the patients had undergone ENT or bariatric surgery, nor used CPAP or oral appliances, even for a trial period. Inclusion required valid anthropometric and comorbidity data for both evaluations. All patients signed an informed consent before the laboratory evaluations and the study was approved by the Faculty of Medicine Ethics committee.

Cardiometabolic comorbidities were extracted from the individual medical records and classified as diabetes (ICD code 250), hypertension (code 401), and cardiovascular diseases (codes 402–459). In Israel, all citizens are insured through one of four national Health Maintenance Organizations (HMOs), each of which maintains a centralized, cumulative electronic medical record (EMR). This EMR integrates all medical encounters—including primary care visits, specialist consultations, hospitalizations, laboratory and imaging results, and diagnoses—regardless of where the care was delivered. Therefore, the data represent the complete diagnostic history of each patient.

For each PSG evaluation, demographic, anthropometric, and sleep parameters were recorded, including Apnea-hypopnea index (AHI)), BMI, sleep efficiency (SE), sleep latency (SL), percentage of REM sleep, percentage of stage 3–4 sleep, percentage of time with arterial oxygen saturation below 91% (SAT < 91%), and the nadir of nocturnal oxygen saturation (nadir SpO₂). Apnea severity was quantified using the A**HI** derived from full PSG and categorized according to standard criteria as none (< 5 events/hour), mild (5–14.9 events/hour), moderate (15–29.9 events/hour), or severe (≥ 30 events/hour). Categorical worsening was defined a priori as progression from AHI < 15 to AHI ≥ 15 events/hour between the first and second studies, reflecting transition to at least moderate OSA. As a sensitivity analysis, worsening was alternatively defined as progression to severe OSA, operationalized as a transition from AHI < 30 to AHI ≥ 30 events/hour at follow-up.

ΔAHI, ΔBMI, ΔSAT < 91%, and Δ nadir SpO₂ were calculated as the differences between the second (T2) and first (T1) evaluations. Incident comorbidities were defined as new-onset diabetes, hypertension, or cardiovascular disease at the second evaluation among patients without these conditions at T1.

### Statistical analysis

Continuous variables are expressed as mean ± standard deviation. Group differences were assessed using *t*-tests for normally distributed variables or Mann–Whitney tests otherwise, and chi-square tests for categorical variables. Multiple linear regression models were used to predict ΔAHI based on demographic, anthropometric, and sleep parameters, as a continuous variable. Logistic regression models were used to identify predictors of ΔAHI as categorized variable and new-onset cardiometabolic morbidities. Model performance was evaluated using area under the curve (AUC).

## Results

### Apnea severity

Table 1 presents the mean age, AHI, BMI, SL, SE, total sleep time, SAT < 91% and nadir SpO_2_, for men and women, for the first and second evaluations. Comparing the evaluations reveals that AHI increased and nadir SpO_2_ decreased significantly for both men (Δ = +2.03, *p* < 0.001; −1.41%, *p* < 0.01) and women (Δ = +3.86, *p* < 0.001; −2.92%, *p* < 0.008). There was a parallel increase in BMI (men Δ = 0.25, *p* < 0.02; women Δ = 1.73, *p* < 0.0001). The differences in AHI, nadir saturation, and BMI between the two evaluations were significantly correlated for both men (*r* = 0.24, *p* < 0.0001;−0.15, *p* < 0.001, respectively) and women (*r* = 0.24, *p* < 0.008; −0.22, *p* < 0.01, respectively).

**Table 1:** Comparison of sleep and physiological variables for men and women in the first and second tests

Variable	Mean T1	SD T1	Mean T2	SD T2	*p*
**Men**					
Age	50.60	12.33	55.03	12.49	**0.0001**
BMI	30.68	5.05	30.94	5.25	**0.02**
SL	31.05	29.16	31.51	32.48	0.80
REM L	102.20	61.90	107.04	65.23	0.22
TST	413.25	49.12	410.06	61.69	0.34
SE	78.11	12.32	77.12	13.67	0.15
AHI	23.92	15.17	25.95	15.54	0.0003
%SAT < 91%	11.08	19.55	11.94	18.57	0.33
NadirSpO_2_%	82.66	9.86	81.25	81.25	**0.013**
%STAGE 3–4	13.49	8.90	12.02	9.19	**0.003**
%REM	18.62	7.10	18.57	6.68	0.89
**Women**					
Age	51.54	10.59	55.59	10.55	0.0001
BMI	32.91	6.81	34.64	7.31	**0.001**
SL	37.92	33.24	43.28	47.26	0.79
REM L	112.99	57.87	120.55	76.15	0.66
TST	415.93	53.95	402.88	69.18	0.25
SE	76.50	13.06	71.83	18.06	0.19
AHI	20.85	15.53	25.60	16.41	**0.0001**
%SAT < 91%	8.21	18.00	8.11	17.18	0.79
NadirSpO_2_%	84.39	9.07	81.47	11.29	**0.008**
%STAGE 3–4	17.39	8.50	12.49	9.06	**0.0007**
%REM	17.72	6.24	19.76	7.44	0.21

Values are mean ± SD. BMI, body mass index; SL, sleep latency; REM L, REM latency; TST, total sleep time; SE, sleep efficiency; AHI, apnea–hypopnea index; %SAT < 91%, percentage of time oxygen saturation < 91%; nadir SpO_2,_ nadir of nocturnal saturation. Paired t-tests were used

Categorizing AHI to severity categories revealed that at baseline, most participants of both sexes had moderate or severe OSA, with women showing a higher proportion of mild disease and men a higher proportion of severe disease (Table 2). Although the baseline severity distribution differed modestly by sex, this difference only bordered on statistical significance (χ², *p* = 0.07). Clinically significant worsening, progression from AHI < 15 to ≥ 15 events/hour, occurred in 49 of 144 men (36.8%) and 17 of 48 women (35.4%) who had AHI < 15 at baseline. In a sensitivity analysis, defining worsening as progression to severe OSA (AHI < 30 to ≥ 30 events/hour), worsening was observed in 58 of 304 men (19.1%) and 14 of 97 women (14.4%) who had AHI < 30 at baseline.

**Table 2:** Obstructive sleep apnea severity categories by sex at first and second evaluations

	No OSA (< 5)	Mild (5–14.9)	Moderate (15–29.9)	Severe (≥ 30)
Men – T1	5 (1.2%)	128 (30.5%)	171 (40.8%)	115 (27.4%)
Men – T2	5 (1.2%)	106 (25.3%)	160 (38.2%)	148 (35.3%)
Women – T1	0 (0.0%)	48 (40.3%)	49 (41.2%)	22 (18.5%)
Women – T2	2 (1.7%)	31 (26.1%)	54 (45.4%)	32 (26.9%)

Percentages are calculated within sex. T1 = baseline PSG; T2 = follow-up PSG

Using multivariable linear regression to identify predictors of worsening apnea severity, modeled as a continuous change in AHI, we found that worsening over time was independently associated with greater weight gain between evaluations (β = 1.13 AHI units per kg/m² increase, 95% CI 0.78–1.48; *p* < 0.001), more severe baseline hypoxemia (β = 0.076 AHI units per 1% increase in time with SpO₂ < 91%, 95% CI 0.03–0.13; *p* = 0.0027), and longer sleep latency at the first test (β = 0.042 AHI units per 1-minute increase, 95% CI 0.01–0.08; *p* = 0.021). In contrast, higher baseline AHI was independently associated with a smaller subsequent increase in AHI (β = −0.286 per AHI unit, 95% CI − 0.35 to − 0.23; *p* < 0.001), consistent with regression-to-the-mean effects. Sex, age, and duration between tests were not independently associated with ΔAHI (Table 3).

**Table 3:** Multivariable linear regression predicting change in AHI

Predictor	β coefficient	95% CI	*p*-value
Age at first test (years)	−0.016	−0.089 to 0.058	0.678
Sex	−0.44	−2.54 to 1.67	0.684
BMI at first test (kg/m²)	0.313	0.146 to 0.479	**0.00024**
ΔBMI	1.130	0.781 to 1.479	**< 0.001**
AHI at first test	−0.286	−0.347 to −0.225	**< 0.001**
% SAT < 91%	0.076	0.027 to 0.126	**0.0027**
Years between tests	0.109	−0.220 to 0.437	0.516
Sleep latency	0.042	0.005 to 0.079	**0.027**
Sleep efficiency	0.055	−0.033 to 0.144	0.220
Total sleep time	0.003	−0.017 to 0.022	0.768

β coefficients represent the change in ΔAHI per unit increase in the predictor. ΔBMI represents the change in BMI between the two tests. Sleep variables from the first evaluation

Logistic regression analysis revealed that both progression from AHI < 15 to ≥ 15 and from AHI < 30 to ≥ 30 was independently predicted only by BMI at the first test (OR – 1.05, 95%CI 1.00–1.13, p = 0.04; OR – 1.06, 95%CI 1.01–1.12, p = 0.01, respectively).

## Comorbidities

### Distribution of cardiometabolic comorbidities

The distribution of cardiometabolic comorbidities at baseline and the incidence of new diagnoses during follow-up are summarized in Table 4. At baseline, hypertension was present in 37.2% of men and 43.7% of women, while diabetes was present in 16.9% of men and 26.9% of women. Other cardiovascular diagnoses, including prior myocardial infarction, angina pectoris, cardiac dysrhythmias, heart failure, and cerebrovascular disease, were less prevalent but occurred more frequently in men than women. During follow-up, incident hypertension occurred in 11.0% of men and 10.9% of women, and incident diabetes developed in 8.6% of men and 13.8% of women. Incident cardiovascular disease events were less common overall but included new diagnoses across several cardiovascular categories in both sexes.

**Table 4:** Hypertension, Diabetes, and cardiovascular diagnoses at baseline and incidental cases at Follow-up, by sex

Diagnosis	Men (*N* = 419)		Women (*N* = 119)	
	Baseline	Incident	Baseline	Incident
**Hypertension**	156 (37.2%)	46 (11.0%)	52 (43.7%)	13 (10.9%)
**Diabetes**	71 (16.9%)	30 (8.6%)	32 (26.9%)	12 (13.8%)
**Cardiovascular diseases**				
Old myocardial infarction	22 (5.3%)	3 (0.7%)	0 (0.0%)	1 (0.8%)
Angina pectoris	39 (9.3%)	18 (4.3%)	6 (5.0%)	2 (1.7%)
Chronic pulmonary heart disease	2 (0.5%)	3 (0.7%)	0 (0.0%)	0 (0.0%)
Cardiac dysrhythmias	14 (3.3%)	6 (1.4%)	2 (1.7%)	4 (3.4%)
Heart failure	7 (1.7%)	2 (0.5%)	1 (0.8%)	1 (0.8%)
Late effects of cerebrovascular disease	9 (2.1%)	7 (1.7%)	5 (4.2%)	1 (0.8%)
Other circulatory system disorders	4 (1.0%)	1 (0.2%)	0 (0.0%)	1 (0.8%)

### Predictors of restricted incident hypertension

Multivariable logistic regression analyses identifying predictors of restricted incident hypertension are presented in Table 5. Older age and higher baseline BMI were independently associated with incident hypertension, with a 5% increase in odds per year of age (OR 1.05, 95% CI 1.02–1.08) and a 9% increase in odds per kg/m² of baseline BMI (OR 1.09, 95% CI 1.02–1.16). Longer duration between PSG evaluations was also a strong independent predictor (OR 1.29 per year, 95% CI 1.16–1.45). Sex, baseline AHI, ΔAHI, Δ BMI, sleep latency, sleep efficiency, and total sleep time were not independently associated with incident hypertension. The model demonstrated good discrimination (C-statistic = 0.76).

**Table 5:** Predictors of restricted incident hypertension

Predictor	Odds Ratio (OR)	95% CI	*p*-value
Sex	1.02	0.46–2.25	0.963
Age at first test (years)	1.05	1.02–1.08	**0.003**
BMI at first test (kg/m²)	1.09	1.02–1.16	**0.007**
ΔBMI (kg/m²)	1.02	0.89–1.16	0.790
AHI at first test	1.01	0.98–1.03	0.551
ΔAHI	1.01	0.98–1.04	0.418
Sleep latency	0.99	0.97–1.00	0.177
Sleep efficiency	1.00	0.97–1.04	0.896
Total sleep time	1.00	0.99–1.01	0.988
Years between tests	1.29	1.16–1.45	**< 0.001**

Odds ratios (OR) and 95% confidence intervals (CI) were estimated using multivariable logistic regression. ΔBMI and ΔAHI represent the change between the first and second tests. Sleep variables from the first evaluation

C-statistic = 0.76

### Predictors of restricted incident cardiovascular disease

Predictors of restricted incident cardiovascular disease are shown in Table 6. In contrast to hypertension, older age (OR 1.05 per year, 95% CI 1.01–1.08), higher baseline apnea severity (OR 1.03 per AHI unit, 95% CI 1.00–1.05), lower sleep efficiency (OR 0.96 per 1% increase, 95% CI 0.93–0.99), and longer duration between studies (OR 1.15 per year, 95% CI 1.01–1.30) were independently associated with incident cardiovascular disease. Baseline BMI, ΔBMI, sex, and ΔAHI were not significant predictors. Model discrimination was acceptable (C-statistic = 0.75).

**Table 6:** Predictors of restricted incident cardiovascular disease

Predictor	Odds Ratio (OR)	95% CI	*p*-value
Sex	0.96	0.41–2.25	0.924
Age at first test (years)	1.05	1.01–1.08	**0.006**
BMI at first test (kg/m²)	0.99	0.93–1.06	0.840
ΔBMI (kg/m²)	0.99	0.85–1.15	0.914
AHI at first test	1.03	1.00–1.05	**0.027**
ΔAHI	0.99	0.95–1.02	0.424
Sleep efficiency	0.96	0.93–0.99	**0.006**
Total sleep time	1.01	1.00–1.02	0.130
Sleep latency	1.00	0.99–1.01	0.879
Years between tests	1.15	1.01–1.30	**0.031**

Odds ratios (OR) and 95% confidence intervals (CI) estimated using multivariable logistic regression. ΔBMI and ΔAHI represent the change between the first and second tests. Sleep variables from the first evaluation

C-statistic = 0.75

### Predictors of restricted incident diabetes

Results of multivariable logistic regression identifying predictors of restricted incident diabetes are presented in Table 7. Higher baseline BMI was independently associated with incident diabetes (OR 1.15 per kg/m², 95% CI 1.05–1.25), as was longer duration between polysomnographic evaluations (OR 1.24 per year, 95% CI 1.07–1.43). Sex, baseline apnea severity, ΔAHI, ΔBMI, and sleep continuity measures were not independently associated with incident diabetes. The diabetes model demonstrated very good discrimination (C-statistic = 0.86).

**Table 7:** Predictors of restricted incident diabetes

Predictor	Odds Ratio (OR)	95% CI	*p*-value
Sex	0.92	0.46–1.83	0.81
Age at first test (years)	1.01	0.98–1.05	0.55
BMI at first test (kg/m²)	1.15	1.05–1.25	**0.002**
ΔBMI (kg/m²)	1.09	0.91–1.30	0.37
AHI at first test	0.99	0.96–1.02	0.42
ΔAHI	1.00	0.96–1.04	0.96
Sleep efficiency	0.99	0.95–1.04	0.76
Total sleep time	1.01	1.00–1.02	0.29
Sleep latency	1.00	0.98–1.02	0.95
Years between tests	1.24	1.07–1.43	**0.004**

Odds ratios (OR) and 95% confidence intervals (CI) estimated using multivariable logistic regression. ΔBMI and ΔAHI represent the change between the first and second tests. Sleep variables from the first evaluation

C-statistic = 0.86

### Comparison of predictors across outcomes

A comparison of independent predictors across cardiometabolic outcomes is summarized in Table 8. Age and duration between studies were consistently associated with incident hypertension, diabetes, and cardiovascular disease. Baseline BMI predicted incident hypertension and diabetes but not cardiovascular disease, whereas baseline apnea severity and sleep efficiency were uniquely associated with incident cardiovascular disease. Sex was not an independent predictor in any of the adjusted models. Replacing AHI with oxygen saturation metrics did not change the results in any of the models.

**Table 8:** Comparison of predictors across outcomes

Predictor	HT (OR, *p*)	DM (OR, *p*)	CVD (OR, *p*)
Age	1.05, *p* = 0.003	NS	1.05, *p* = 0.006
BMI (first test)	1.09, *p* = 0.007	1.15, *p* = 0.002	NS
ΔBMI	NS	NS	NS
AHI (first test)	NS	NS	1.03, *p* = 0.027
ΔAHI	NS	NS	NS
Sleep efficiency	NS	NS	0.96, *p* = 0.006
Years between tests	1.29, *p* = 0.001	1.24, *p* = 0.004	1.15, *p* = 0.03
Sex	NS	NS	NS

*OR* Odds ratio; *HT* hypertension; *DM* diabetes mellitus; *CVD* cardiovascular disease; *NS* not statistically significant (*p* ≥ 0.05). For all analyses C-statistic > 0.75

## Discussion

Whether obstructive sleep apnea (OSA) is a progressive disorder is of considerable clinical importance. Given its high prevalence and the observation that many patients—particularly those with mild OSA—either fail to adapt to or decline treatment [10, 11], determining whether the syndrome’s severity evolves and understanding the associated clinical consequences has major implications for patient management.

This longitudinal study offers new insights into the natural history of untreated OSA and its cardiometabolic consequences over a mean follow-up of nearly four years. The present findings demonstrate that, in the absence of therapy, OSA severity generally worsens with time, accompanied by progressive increases in BMI and the emergence of cardiometabolic comorbidities. These results emphasize the importance of continuous monitoring and early intervention, even for patients who refuse active treatment.

Consistent with prior longitudinal and population-based studies—such as the Wisconsin Sleep Cohort [4] and the Sleep Heart-Health Study (SHHS) and the work of Inoue et al. [2]—we found that weight gain and higher baseline BMI were strong predictors of worsening apnea severity. In agreement with Fisher et al. [3], changes in BMI were significantly correlated with changes in AHI and with the nadir of nocturnal hypoxemia in both men and women, reinforcing the central role of obesity in OSA progression. In the Wisconsin Sleep Cohort, Peppard and colleagues [4] showed that modest weight gain was associated with substantial increases in AHI, whereas weight loss led to meaningful improvement in apnea severity, underscoring the sensitivity of continuous apnea metrics to physiologic change.

In the present longitudinal cohort of untreated patients undergoing repeat PSGs, apnea severity frequently worsened over time, but the determinants of progression differed depending on how severity was modeled. When the change in AHI was analyzed as a continuous outcome, worsening was independently associated with higher baseline BMI, weight gain, greater baseline hypoxemia, and longer sleep latency. These findings are consistent with population-based longitudinal studies demonstrating that obesity is a dominant driver of apnea progression, but they also highlight additional physiologic contributors that may be obscured when apnea severity is examined categorically.

Beyond obesity, the independent association between baseline hypoxemia and worsening AHI aligns with prior SHHS analyses demonstrating that measures of nocturnal oxygen burden capture aspects of disease severity not fully reflected by event frequency alone [12]. Similarly, the association between longer sleep latency and worsening AHI supports emerging evidence that impaired sleep initiation and heightened arousal propensity contribute to ventilatory instability and progressive sleep-disordered breathing. Experimental and clinical studies have shown that individuals with lower arousal thresholds experience more frequent respiratory events due to premature arousal before airway-stabilizing ventilatory drive can accumulate [13, 14]. These findings suggest that sleep quality and arousal physiology are integral components of apnea progression when assessed using continuous measures.

In contrast, logistic regression analyses examining progression across conventional severity thresholds (AHI < 15 to ≥ 15, and AHI < 30 to ≥ 30) identified baseline BMI as the primary predictor, while sleep-related variables and hypoxemia were not independently associated. This discrepancy is expected and reflects the threshold-dependent nature of categorical severity classifications. Analyses from both SHHS and WSC have shown that baseline apnea severity and proximity to diagnostic cut points strongly influence categorical transitions, whereas smaller but clinically meaningful changes may not result in reclassification unless thresholds are crossed [4, 15]. As a result, categorical analyses may underestimate gradual physiologic worsening and are less sensitive to the multifactorial contributors to disease progression.

### Apnea severity and cardiometabolic comorbidities

The distinction between continuous and categorical apnea measures was also evident in analyses of cardiometabolic outcomes. In adjusted models, incident hypertension and diabetes were independently associated with age, baseline BMI, and duration of follow-up, but not with baseline AHI, ΔAHI, or hypoxemia. These findings are consistent with prior SHHS and WSC studies showing that associations between OSA and hypertension or diabetes are substantially attenuated after accounting for obesity and weight change [16, 17]. In contrast, incident cardiovascular disease was independently associated with baseline apnea severity and reduced sleep efficiency, supporting a distinct mechanistic pathway linking sleep-disordered breathing and sleep fragmentation to cardiovascular risk. SHHS investigators have demonstrated independent associations between AHI, nocturnal hypoxemia, arousal burden, and incident atrial fibrillation, heart failure, and stroke [18–20]. Several studies reported that the combination of insomnia and OSA constitutes a greater cardiovascular risk than OSA alone [21, 22]. Our findings extend these observations by showing that sleep efficiency and apnea severity remain predictive of cardiovascular disease even when obesity-related factors are considered.

### Clinical implications

Taken together, these results suggest that continuous measures of apnea severity provide a more sensitive framework for capturing disease progression and its physiologic determinants, whereas categorical severity classifications primarily reflect threshold-based transitions driven by obesity. From a clinical perspective, reliance solely on severity categories may underestimate meaningful worsening in untreated patients, particularly those with increasing hypoxemia or impaired sleep quality who have not crossed diagnostic thresholds. Longitudinal assessment incorporating continuous apnea metrics and sleep quality measures may therefore improve risk stratification, inform decisions regarding treatment initiation, and better identify patients at risk for adverse cardiovascular outcomes.

The progression of OSA and its metabolic sequelae observed in our untreated cohort contrasts with outcomes reported among treated patients. Randomized and observational studies have demonstrated that effective CPAP therapy mitigates cardiometabolic risk. For instance, the HIPARCO trial showed significant reductions in 24-hour blood pressure in resistant hypertensive patients after 12 weeks of CPAP use, and long-term cohort studies reported a lower incidence of new-onset hypertension among adherent user [23–25]. Although large randomized trials such as SAVE and RICCADSA failed to show reductions in major cardiovascular events, these results are generally attributed to poor adherence (< 4 h/night) and the inclusion of patients with established cardiovascular disease [26, 27]. Meta-analytic evidence further indicates that CPAP improves insulin sensitivity and glycemic control, but consistent prevention of incident diabetes has not been established, again depending strongly on nightly usage [28, 29]. Collectively, these data suggest that while untreated OSA predisposes patients to progressive cardiometabolic deterioration—as demonstrated in the present study—adequate and sustained treatment can substantially attenuate these risks. The differences between treated and untreated populations highlight the importance of therapeutic adherence and reinforce the need for early identification and engagement of patients who decline or discontinue therapy.

### Strengths and limitations

This study has several notable strengths. First, the use of repeated attended PSGs allowed direct longitudinal assessment of changes in apnea severity using consistent scoring methodology, minimizing measurement variability. Second, modeling apnea progression using both continuous (ΔAHI**)** and categorical severity transitions provided complementary insights into physiologic worsening and clinically defined progression. Third, comprehensive characterization of cardiometabolic comorbidities through longitudinal electronic medical record review enabled robust assessment of incident hypertension, diabetes, and cardiovascular disease. Finally, integration of sleep architecture and sleep continuity measures allowed evaluation of sleep quality as a contributor to apnea progression beyond traditional apnea frequency metrics.

Several limitations should also be considered. The observational design precludes causal inference, and residual confounding cannot be excluded. Although this cohort is relatively large for a clinical longitudinal PSG study, event counts for some cardiovascular outcomes were modest, limiting statistical power and necessitating parsimonious modeling. Participants were untreated by choice, which may introduce selection bias and limit generalizability to patients who accept therapy. In addition, categorical severity transitions are inherently influenced by proximity to diagnostic thresholds, which may underestimate meaningful physiologic worsening captured by continuous measures. Finally, changes in lifestyle factors other than weight (e.g., physical activity or medication use) were not systematically captured.

## Conclusions

In this longitudinal study of untreated obstructive sleep apnea, apnea severity commonly worsened over time, with progression driven by obesity-related factors as well as baseline hypoxemia and impaired sleep initiation when modeled as a continuous outcome. In contrast, progression across conventional severity categories was primarily influenced by baseline BMI and threshold effects, underscoring the limitations of categorical classifications for capturing gradual disease worsening. Cardiometabolic consequences also differed by outcome: hypertension and diabetes were dominated by metabolic risk factors, whereas cardiovascular disease was independently associated with apnea severity and sleep fragmentation. Together, these findings support the use of continuous apnea metrics and sleep quality measures for longitudinal monitoring and risk stratification and suggest that reliance solely on categorical severity thresholds may underestimate clinically relevant progression in untreated patients.

## Data Availability

The datasets analysed during the current study are available from the The Technion Sleep Center on reasonable request.
